# Highly pathogenic Bovine Respiratory Syncytial virus variant in a dairy herd in Italy

**DOI:** 10.1002/vms3.312

**Published:** 2020-06-28

**Authors:** Monica Giammarioli, Piermario Mangili, Alex Nanni, Ilaria Pierini, Stefano Petrini, Silvia Pirani, Paola Gobbi, Gian Mario De Mia

**Affiliations:** ^1^ Istituto Zooprofilattico Sperimentale Umbria e Marche “Togo Rosati” Perugia Italy; ^2^ AUSL Romagna ‐ Ambito territoriale Rimini Rimini Italy

**Keywords:** bovine respiratory syncytial virus, high mortality, phylogenetic analysis

## Abstract

Bovine respiratory syncytial virus (BRSV) is an economically significant pathogen in cattle production worldwide. Usually, it is detected in outbreaks of respiratory disease, most often during the winter period. During the middle of October 2018, a serious outbreak of respiratory disease occurred in a cattle farm comprising about 300 heads located in Central Italy. The herd was affected by a severe flu‐like syndrome unresponsive to any antibiotic treatment. Within 3 weeks, 39 adult animals died, and 12 abortions occurred. Direct and indirect laboratory tests were performed to detect the main pathogens causing the respiratory disease of the affected cattle. The results of laboratory investigations provided evidence of an acute and severe BRSV syndrome characterized by unusual mortality. In order to investigate the molecular underpinnings of this syndrome, phylogenetic analysis of the BRSV strain detected from the outbreak was carried out. The sequence analysis showed that the strain was genetically divergent from BRSV strains previously identified in Italy, as it showed high sequence similarity of more than 97% with strains isolated during a major BRSV epizootic that occurred in Sweden, Norway and Denmark during 2010–2011. The infection of the herd in Italy with this BRSV strain was likely due to the introduction of animals imported into Italy from abroad.

## Graphical abstract

Bovine respiratory syncytial virus (BRSV) is an economically significant pathogen in cattle production. In Italy, although respiratory problems due to BRSV are frequently described, major outbreaks have not yet been reported. Here, we report an outbreak caused by a BRSV strain with high mortality that occurred in a dairy farm in Central Italy; this strain shows a sequence similarity of >97% to the strains isolated during a major BRSV epizootic that occurred in Sweden and Norway during 2010–2011. Epidemiological analysis indicated that recently introduced heifers from Germany were the likely carriers of the virus.

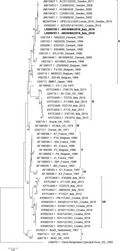

## 1 INTRODUCTION

Bovine respiratory syncytial virus (BRSV) belongs to the genus *Orthopneumovirus*, within the *Pneumoviridae* family; it is responsible for an acute respiratory disease syndrome in beef and dairy calves, particularly during winter periods. Globally circulating BRSV has a significant negative impact on the cattle industry due to economic losses because of the associated morbidity, mortality, costs of treatment and prevention, loss of production and reduced carcass value (Schneider, Tait, Busby, & Reecy, 2009; Valarcher, Schelcher, & Bourhy, 2000).

Bovine respiratory syncytial virus is an enveloped virus, and its genome contains 10 mRNAs, which are transcribed and translated into 11 viral proteins (Valarcher & Taylor, 2007). Viral glycoprotein (G protein) has been suggested to be the major viral antigen that induces host immune response and confers a good level of protection. Variation of some G protein regions is believed to be antigenically important (Elvander et al., 1998; Prozzi et al., 1997). Biological significance of antigenic variation might be relevant to the efficacy of vaccines (Prozzi et al., 1997). The gene encoding G protein, as well as those encoding nucleoprotein (N) and fusion (F) protein have been used as targets of phylogenetic and molecular epidemiological studies (Valarcher & Taylor, 2007). Based on this analysis, eight subgroups (I–VIII) were assigned to represent both the temporal and geographical distribution of known isolates (Bertolotti, Giammarioli, & Rosati, 2018; Krešić et al., 2018; Valarcher & Taylor, 2007).

Laboratory investigations and related phylogenetic studies of BRSV have been previously reported in Denmark, Sweden, Norway, Czech Republic, Poland, Croatia, Italy, USA, Brazil, Japan, Britain (Almeida et al., 2016; Beaudeau, Björkman, Alenius, & Frössling, 2010; Bertolotti et al., 2018; Bidokti Medhi et al., 2012; Klem et al., 2013; Klem, Rimstad, & Stokstad, 2014; Kovarcík & Valentová, 2004; Krešić et al., 2018; Nettleton et al., 2003; Sarmiento‐Silva, Nakamura‐Lopez, & Vaughan, 2012; Socha, Larska, & Rola, 2009; Yaegashi, Seimiya, Seki, & Tsunemitsu, 2005). Like other RNA viruses, BRSV has high genetic heterogeneity and a rapid mutation rate, so that several viral subpopulations may originate from the same host (Sarmiento‐Silva et al., 2012). BRSV is characterized by a high rate of sequence evolution, resulting in local genetic differentiation (Valarcher et al., 2000). As a consequence, the level of protection provided by different vaccine strains has to be re‐evaluated. Moreover, genetic characterization showed a strict geographic correlation between virus variants and the emergence of new variants in Europe (Bidokti Medhi et al., 2012). There is limited information about the similarity and evolutionary relationship among BRSV isolates, particularly in those isolated from an Italian cattle population. Two different clades have been identified so far in Italy, namely subgroup III and subgroup VII (Bertolotti et al., 2018). In this study, we describe identification and genetic characterization of a BRSV strain that was responsible for unusual mortality and relevant morbidity during an outbreak in a dairy farm in Central Italy.

## 2 MATERIAL AND METHODS

### 2.1 Outbreak and sample collection

In the middle of October 2018, a serious outbreak of respiratory disease occurred in a cattle farm of about 300 heads of Friesian breed, located in Central Italy. This breed is reared predominantly for milk production, and to a lesser extent, fattening of beef calves. The non‐vaccinated herd was affected by severe and unusual flu‐like manifestations, including dyspnoea, anorexia, hyperthermia, loss of appetite, poor mobility and suffering, drastic reduction in milk production and severe respiratory symptoms, which were not relieved by any antibiotic therapy. Within 3 weeks, 39 adult animals died, and 12 abortions occurred at various gestational ages, especially in the animals with more severe symptoms. Unfortunately, the aborted fetuses were not submitted to laboratory for abortion diagnosis, and consequently the potential causes were not identified. Necropsies were performed by an on‐site veterinary practitioner during this 3‐week period as mortality occurred. Samples of the upper respiratory tract, lungs and nasopharyngeal swabs of the 35 animals that died with clinical respiratory disease were sent to the laboratory for pathological and laboratory investigations for the presence of the main pathogens causing the respiratory disease of cattle. In addition, serum samples were randomly collected from 20 convalescent animals to detect antibodies against the main viral agents of bovine respiratory disease complex.

### 2.2 Laboratory diagnostic tests

Laboratory diagnostic tests were carried out for bovine parainfluenza type 3 virus (BPIV‐3), bovine coronavirus (BCoV), bovine respiratory syncytial virus (BRSV), bovine viral diarrhoea virus (BVDV), infectious bovine rhinotracheitis (IBR), and major bacterial agents that could have caused respiratory pathologies in cattle namely *Mycoplasma* spp., *Mannheimia haemolytica*, *Histophilus somni* and *Pasteurella multocida*.

Virological investigations were performed by conventional and real‐time PCR/RT‐PCR (Decaro et al., 2008; OIE Manual of Diagnostic Tests & Vaccines for Terrestrial Animals, 2019a, 2019b; Thonur et al., 2012), bacteriological investigations for *Mycoplasma* spp. were carried out by conventional PCR (Lierz et al., 2007), whereas the presence of *Mannheimia haemolytica,* *Histophilus somni* and *Pasteurella multocida* was investigated by direct seeding on differential growth medium (Timsit et al., 2017). Serological investigations were conducted using commercial Elisa kits: Prima Check^®^ PI‐3 Ab (Agrolabo Spa); Svanovir^®^ BCV Ab (Boeringer Ingelheim Svanova); Prima Check^®^ BRSV (Agrolabo Spa); Ab IDEXX^®^ BVDV p80 Ab and IDEXX^®^ IBRgB X3 (IDEXX Laboratories) following the manufacturer's instructions.

### 2.3 Sequencing and phylogenetic analysis

Total RNA was extracted using a QIAamp Viral RNA mini kit (Qiagen) and used as a template for the amplification of a 541‐bp region encoding the G protein. Amplification was performed by using a one‐step RT‐PCR kit (Qiagen) following the manufacturer's instructions, applying a previously published nested protocol (Vilcek, Elvander, Ballagi‐Pordány, & Belák, 1994). In addition, a fragment of a 731‐bp region encoding the N protein was amplified and sequenced to confirm the subgroup association (Bertolotti et al., 2018; Valarcher et al., 2000). Sense and antisense strands of two PCR‐positive BRSV samples were sequenced in three independent reactions for each isolate. Sequence data were analysed using SeqManPro program from DNAStar package. Clustal X.2 was used to align the sequences with respect to the amino acid‐coding frame with a set of reference sequences from GenBank, including representatives of the eight subgroups proposed previously (Bertolotti et al., 2018; Krešić et al., 2018; Valarcher et al., 2000). BioEdit sequence alignment editor version 7.0.5.2, was used for editing (Hall, 1999). The phylogenetic tree was drawn by applying the Kimura 2‐parameter model with gamma distribution (K2 + G), using MEGA v.7. The robustness of the clusters was assessed by performing 10,000 bootstrap replicates, and branches with bootstrap values exceeding 70% were grouped together (Kumar, Stecher, & Tamura, 2016).

## 3 RESULTS AND DISCUSSION

Overall, the pathological examination showed tracheal congestion, fibro‐purulent bronchopneumonia, marked oedema and interstitial emphysema characterized by consolidation of the apical and middle lobes of the lungs. The results of laboratory investigation are presented in Table 1. Clinical specimens from all the 35 animals analysed were positive for BRSV; in addition, three lungs were positive for *Mycoplasma* spp., and two lungs for *Mannheimia haemolytica*. Serological investigations revealed the presence of antibodies against BPIV‐3 in four out of 20 animals, and against BRSV in all 20 animals. Based on the clinical and laboratory findings, the diagnosis was strongly in favour of acute BRSV syndrome. Regarding abortions, infectious agents associated with abortion in cattle were not investigated. On the other hand, the abortifacient role of BRSV has never been demonstrated, so we assume that the respiratory illness might have caused abortion secondary to the systemic infection, as it can occur for any disease which results in a sick cow and a high fever, although that can only be speculated.

**TABLE 1:** Laboratory results of analysed samples from both dead and convalescent animals

Pathogen	Sample type^a^			
	Lung	Upper respiratory tract	Rhinopharyngeal swab	Serum
BCoV	0/35^b^	0/35	0/35	0/20
BoHV‐1	0/35	0/35	0/35	0/20
BVDV	0/35	0/35	0/35	0/20
BRSV	35/35	35/35	35/35	20/20
BPIV‐3	0/35	0/35	0/35	4/20
*Histophilus somni*	0/35	0/35	0/35	nd
*Mannheimia haemolytica*	2/35	nd	0/35	nd
*Mycoplasma* spp.	3/35	nd	0/35	nd
*Pasteurella multocida*	0/35	nd	0/35	nd

Abbreviation: nd, not determined.

^a^ Genome detection from clinical specimens was performed using RTqPCR and conventional PCR; serological detection was performed using commercial ELISAs: Prima Check^®^ PI‐3 Ab (Agrolabo), Svanovir^®^ BCV Ab (Boeringer Ingelheim Svanova); Prima Check^®^ BRSV (Agrolabo); Ab IDEXX^®^ BVDV p80 Ab and IDEXX^®^ IBRgB X3 (IDEXX).

^b^ Positive detection ratio.

**FIGURE 1:**
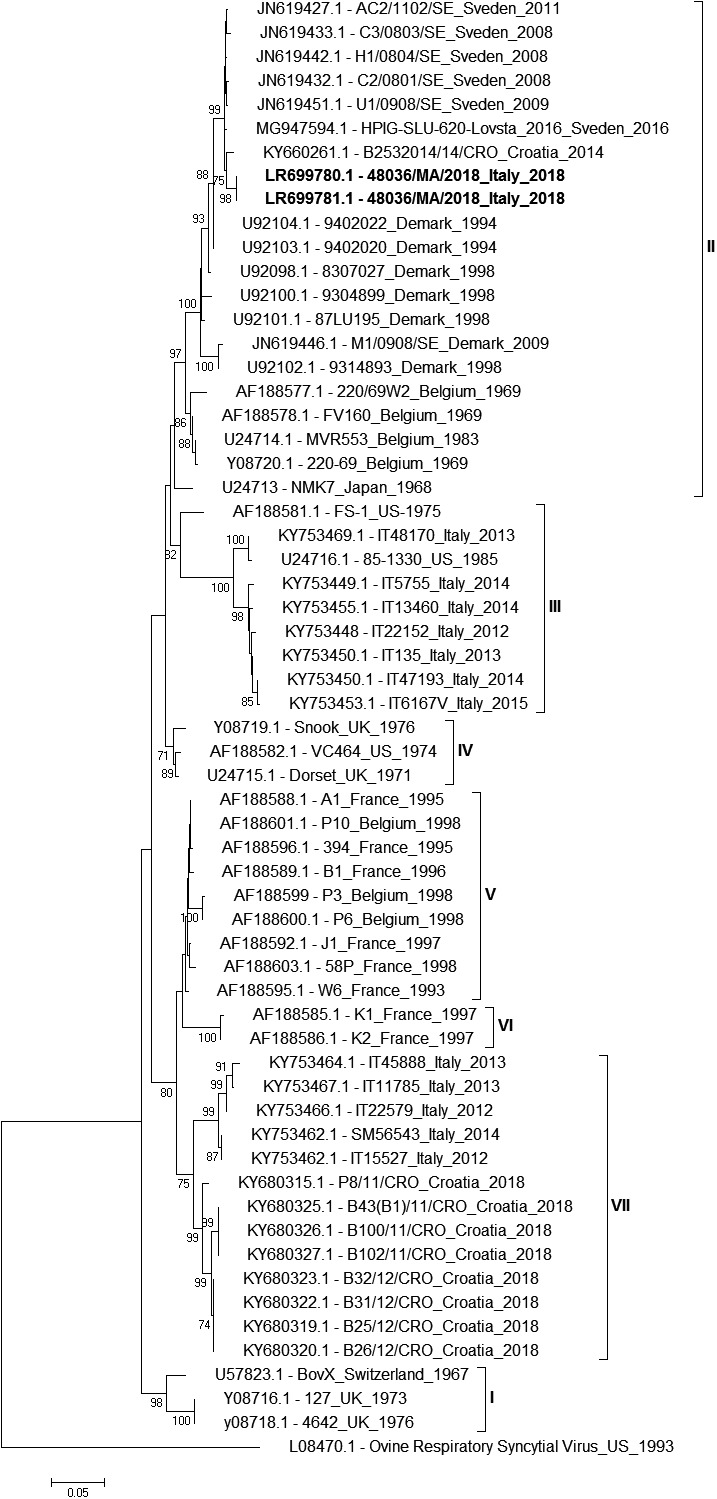
Phylogenetic tree of bovine respiratory syncytial virus G gene partial sequence. The isolates sequenced in this work are labelled in bold. Bar indicates the number of substitutions per site

The sequence analysis conducted on two PCR‐positive BRSV samples (Figure 1), showed that this virus was genetically divergent from the BRSV strains previously identified in Italy (Bertolotti et al., 2018). The BRSV strain, namely 48036/MA/2018 detected in this study clustered together with viral strains identified in Sweden and Denmark during outbreaks of respiratory illness with unusual mortality, which were also responsible for extensive forms of the disease in Sweden and Norway between 2010 and 2011 (Bidokti Medhi et al., 2012). The virus displayed an identity of 97.3% with isolates from Sweden. To confirm the grouping suggested by the G protein sequence, we also analysed a 731‐bp fragment encoding the N protein. The resulting phylogenetic tree showed that the studied viral strain clustered in the same phylogenetic branches as those identified in the G‐protein‐based tree (data not shown). The sequences analysed were deposited in GenBank (accession numbers: LR699780.1, LR699781.1).

We reported an outbreak with high mortality that occurred in a dairy farm in Central Italy and was attributed to a BRSV strain never described in the country. This strain had extremely negative effects on herd health and animal performance, as it had induced a rather high mortality and severe signs of respiratory disease. In few samples from lungs BRSV was detected together with either *Mycoplasma* spp. or *Mannheimia haemolytica*.

After the diagnosis, an emergency vaccination plan was set up with a live attenuated BRSV vaccine, which was administered intra‐nasally. Nevertheless, the animals continued to die. Live and inactivated vaccines against BRSV are generally used, but their efficiency has been questioned (Valarcher et al., 2000). In mid‐November, the health status of survivors stabilized, and milk production partially recovered. During the epidemiological investigation, it was established that in the end of September 2018, 22 Friesian cattle purchased from Germany had been introduced into the farm. They were already in lactation and enjoyed good health. About a couple of weeks after their introduction into the farm, one of these subjects presented a mild self‐resolving respiratory syndrome 10 days before the beginning of the disease outbreak.

The BRSV strain characterized in this study did not cluster together with the viruses previously identified in Italy, but it belonged to subgroup II with isolates collected in Sweden, Norway and Denmark during a major BRSV epizootic that occurred during 2010–2011. As a result, in Italy, at least three distinct groups of BRSV have been detected so far, with our strain representing a group with particularly high virulence. The recent introduction to the herd of 22 heifers that came from Germany, which has frequent commercial exchanges with northern European countries, likely explains the circulation of this new BRSV strain on the farm. Viruses from areas where vaccination is heavily used show frequent amino acid changes, which may lead to the emergence of strains that can potentially evade the immunity conferred by the vaccine. Our study also raises an important question about the adequacy of the available diagnostic tools and implications for control programs. Continuous investigation and molecular characterization of positive samples collected in the country are useful tools for updating knowledge on BRSV epidemiology and evolution of the virus during the time. Moreover, we could notice the emergence of new viral variants that may escape vaccination protection. The successful control and eradication of a virus is possible when the epidemiology of the virus infection is clear and accurate diagnostic methods are available. In addition, an update of the vaccines is also recommended.

## CONFLICT OF INTEREST

The authors declare no conflicts of interest.

## AUTHOR CONTRIBUTION

**Monica Giammarioli**: conceptualization; data curation; formal analysis; writing‐original draft. **Piermario Mangili and Alex Nanni**: investigation. **Ilaria Pierini, Silvia Pirani and Paola Gobbi**: methodology; software; validation. **Stefano Petrini**: methodology; supervision; validation. **Gian Mario De Mia**: supervision; writing‐review & editing.

## ETHICS STATEMENT

The authors confirm that the ethical policies of the journal, as noted on the journal's author guidelines page, have been adhered to and the appropriate ethics review committee approval has been received.

## Data Availability

The data that support the findings of this study are openly available in NCBI at www.ncbi.nlm.nih.gov, reference numbers LR699790.1, LR699781.1.
